# Neuroplasticity of Language Networks in Aphasia: Advances, Updates, and Future Challenges

**DOI:** 10.3389/fneur.2019.00295

**Published:** 2019-04-02

**Authors:** Swathi Kiran, Cynthia K. Thompson

**Affiliations:** ^1^Sargent College of Health and Rehabilitation Sciences, Boston University, Boston, MA, United States; ^2^Department of Communication Sciences and Disorders, Northwestern University, Evanston, IL, United States; ^3^Department of Neurology, The Cognitive Neurology and Alzheimer's Disease Center, Feinberg School of Medicine, Northwestern University, Chicago, IL, United States

**Keywords:** stroke, aphasia, neuroimaging (anatomic and functional), plasticity, recovery

## Abstract

Researchers have sought to understand how language is processed in the brain, how brain damage affects language abilities, and what can be expected during the recovery period since the early 19th century. In this review, we first discuss mechanisms of damage and plasticity in the post-stroke brain, both in the acute and the chronic phase of recovery. We then review factors that are associated with recovery. First, we review organism intrinsic variables such as age, lesion volume and location and structural integrity that influence language recovery. Next, we review organism extrinsic factors such as treatment that influence language recovery. Here, we discuss recent advances in our understanding of language recovery and highlight recent work that emphasizes a network perspective of language recovery. Finally, we propose our interpretation of the principles of neuroplasticity, originally proposed by Kleim and Jones (1) in the context of extant literature in aphasia recovery and rehabilitation. Ultimately, we encourage researchers to propose sophisticated intervention studies that bring us closer to the goal of providing precision treatment for patients with aphasia and a better understanding of the neural mechanisms that underlie successful neuroplasticity.

## Introduction

Stroke affects 15 million people worldwide and approximately 800,000 people in the United States, with an estimated one third (35%) of stroke survivors left with aphasia in the chronic stage (2). Furthermore, post-stroke aphasia significantly negatively impacts quality of life (3, 4) and has a greater negative effect than other common diseases such as cancer, Alzheimer's and Parkinson's disease (5). It is therefore important that research advances our understanding of ways to alleviate the social isolation and lack of autonomy in individuals with aphasia. With advances in neuroimaging and other technologies, a large literature has emerged in the past two decades focused on the brain and language recovery, adding substantially to what we know, challenging early ideas about aphasia recovery and providing early and promising results for neuroplasticity in chronic stroke survivors. In this paper we review this work, emphasizing that the age-old nature, nurture dichotomy extends to recovery from aphasia.

## What we Know About Language Recovery and the Brain

Animal models of recovery from brain damage as well as early studies focused on sensory and motor learning in a variety of mammalian species have illuminated our understanding of the adaptive capacity of the brain (6, 7). Extension of this work to human brains has shown that neural networks are dynamic constructs which undergo remodeling throughout the lifespan based on experience. Further, when the brain is damaged, as in stroke-induced aphasia, experience is crucial for rewiring of neural networks (8–10).

One of the most important facts about language recovery after stroke is that it is a non-linear process, with differences in recovery processes and patterns associated with the age of the stroke. While it is well–documented that the greatest changes in the neural architecture for language occur in early stages of recovery, neuroplasticity occurs even in chronic aphasia, when neurophysiological repair processes have largely been completed (11). Three epochs of recovery have been identified including two early phases, the acute phase and the subacute, and a third chronic phase (12).

### Early Epochs of Recovery Following Stroke

In the acute recovery period, beginning immediately after stroke lasting several hours, a series of events associated with disruption of neurophysiological and metabolic processes both proximal and distal to the infarct results in detrimental but dynamic changes in the brain. Edema may result in a shift of midline structures, affecting several bilateral cortical, and subcortical regions with associated multiple behavioral deficits. Excitotoxicity, the abnormal release of neurotransmitters such as glutamate in the synapse results in high levels of calcium ions in neurons, which results in cell death. Ultimately, since healthy neurons are deprived of input from destroyed neurons via interruptions in intra- or inter-hemispheric pathways, this cascading process results in additional cell death and transneuronal degeneration. Further, focal infarcts result in hypometabolism in intact but remote regions due to abnormal connections that affects behavior (diaschisis) (13) but there is also evidence that suggests that remote regions show increased activity as a consequence of the stroke (14). Changes in blood flow (perfusion) also result in hypoperfusion in both cerebral hemispheres and particularly in the penumbral (perilesional) region within the first 24 h following stroke onset (15).

The subacute phase begins a few days after stroke and lasts several weeks, during which the brain undergoes several changes enabling spontaneous recovery and repair. In this phase, edema is resolved and several abnormal processes return to more normal state. In addition, as the injured tissue recovers from detrimental events, several brain-repair-related events occur. Synaptogenesis results in new connections that may either involve unmasking of previously latent pathways or formation of new pathways (12, 16). Additionally, if the cell body remains functional, axons and dendrites may regenerate leading to axonal and collateral sprouting, which expand existing synaptic connections and consequent neurogenesis in cortical tissue adjacent, and remote, to the infarcted tissue (17). These processes reach peak levels during this period, resulting in a shift from initial increases in excitation of contralesional tissue due to decreases in inhibition from the lesioned hemisphere, to upregulation of functionally viable brain tissue (12). In the motor recovery literature, several studies have noted functional improvement and concomitant engagement and/or reengagement of both ipsilesional and contralateral connections during this period [see (16) for a review]. With respect to language, Saur (18) found significantly increased contralesional (right) and reduced ipsilesional (left) hemisphere fMRI activation (as compared to neurotypical adults) immediately following stroke onset, followed ~12 days after stroke by an upregulation of spared tissue in regions within left as well as right hemisphere regions homologous to left language areas. By 1-year post-stroke, peak activation decreased in the right hemisphere, shifting to greater activation in the left. The relation between these activation shifts and recovery of function, however, is unclear since the natural history of language recovery and associated neural activation has not been charted across early and chronic phases of recovery. We return to this issue below as it relates to the role of the right hemisphere in language recovery.

### The Chronic Phase of Recovery

The third phase of recovery, and most pertinent to this paper, is the chronic phase, which may span months to years after stroke. Although physiological changes occurring during the repair phase have generally subsided as the brain reaches a stable state, mechanisms that facilitate plasticity (i.e., synaptic sprouting) remain at play and are adaptable to environmental experience. Notably, it is now known that brain changes occur throughout the life span, particularly associated with experience (8, 9, 19). For many stroke survivors with aphasia, treatment improves language abilities and associated neural processing of language several years post-stroke (20). However, not all patients show the same degree of recovery over time. This is partly because, among other reasons that influence recovery, the neural sequela of stroke persists into this phase of recovery (21) and interact with experience.

## Factors That Influence Neural Plasticity in Stroke-Induced Aphasia

**Figure 1:**
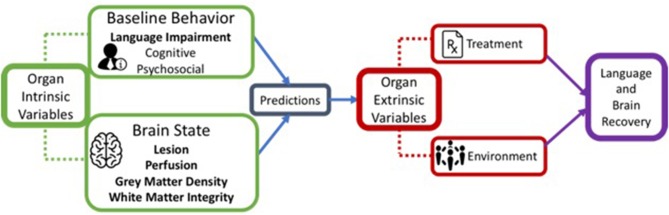
A schematic representation of organism intrinsic variables and organism extrinsic variables that influence language recovery.

There are several factors that influence neural plasticity, including both biological and environmental and, as with any learning system, recovery from aphasia involves a synergistic relation between them (22, 23). We refer to these as *organism intrinsic* and *organism extrinsic* factors. Intrinsic factors consist of behavioral and neural variables related to the stroke itself, including lesion characteristics and patterns of impaired language, whereas extrinsic factors include environmental factors such as treatment (see Figure 1). Recognizing the importance of other intrinsic factors such as personality traits and domain-general cognitive functions (e.g., attention, memory, executive function) as well as extrinsic psychosocial variables (e.g., participation in social and work-related activities, and support systems), here we constrain our discussion to neural and treatment variables.

### Organism Intrinsic (Neural) Variables Associated With Recovery

The neural factors that impact recovery include (but are not limited to) age at the time of stroke, lesion volume and location, vascular pathophysiology, and white matter integrity.

#### Age at the Time of Stroke

The age of the brain at the time of stoke is well-known to impact recovery, in that younger brains are more plastic than older brains. Thus, childhood stroke-induced aphasia has better overall outcomes than adults, although learning and other cognitive skills may remain compromised (24, 25). In adults, the picture is quite different although the stroke-aphasia literature suggests a tendency for older patients to recover less well-than younger patients (26). This may, in part, be related to findings that language and other domain-general cognitive processes shift from being left lateralized to becoming more bilateral in the older brain during the aging process (27). Future research will need to examine whether older adults with aphasia show the same extent of recovery and reorganization as younger adults with aphasia and how/if brain age interacts with other organism internal predictors of recovery.

#### Lesion Volume and Location

The relation between lesion characteristics and recovery of function has been addressed by several research groups. In general, large left hemisphere lesions are typically associated with poorer recovery (28, 29), whereas smaller lesions suggest better language recovery. Presumably, this is because smaller left hemisphere lesions leave a greater volume of tissue available for remapping of the language network, whereas large left hemisphere lesions limit tissue available for reorganization of function. Also, larger lesions are more likely to affect a greater number of language operations as well as domain-general systems engaged for language processing (29). However, the relation between lesion volume and recovery is not straight forward. More recent research suggests that while overall lesion volume may explain the degree of language recovery, the location of the lesion is equally if not more important in predicting recovery (30). In the motor domain, damage to specific descending tracts more accurately predict recovery than overall lesion volume (31) and lesions to the corona radiata, internal capsule, and insula impact functional outcome on the modified Rankin scale to a greater extent than lesion volume alone (32). Likewise, with respect to language recovery, Hillis found that damage to left posterior superior temporal gyrus and fibers that included superior longitudinal fasciculus and arcuate fasciculus negatively influenced the degree of recovery of naming skills (33) in acute and chronic patients with aphasia, and these findings were independent of total lesion volume. Correspondingly, sparing of tissue in left temporal parietal regions, for example, has been associated with improvements in naming resulting from treatment (34, 35).

However, other studies have found no relationships between recovery of naming skills and damage in any left hemisphere region (36). In a different analysis, Skipper-Kallal and colleagues found that total lesion volume in the left hemisphere predicted right hemisphere activation for both overt naming and covert word retrieval (37). However, no relationships were found between naming skills and damage in any given LH region when the authors controlled for total lesion volume and no relationships were observed between naming skills and right anterior activation suggesting that damage to LH IFG regions does not necessite activation in the corresponding RH regions. These results suggest that reorganization of language, while dependent on lesion size and site, may be more complex than the simple reengagement of homologous regions. Another recent study focused on recovery of sentence processing, however, found that although greater left hemisphere lesions within language-specific networks was associated with recovery and concomitant recruitment of right hemisphere neural networks, lesser damage to left domain-general network predicted recovery (38).

It should be noted that while aforementioned studies have specifically examined language processing and recovery, a number of studies have examined specific lesion locations that are associated with specific language impairments. The reader is referred to recent extensive work utilizing Voxel-based Lesion Symptom Mapping (VLSM) (39–43) and other quantitative approaches (39, 44) that provide insight into gray matter regions that are crucial for language functions by establishing the relationship between damaged/infarcted tissue and impairments in naming, semantic processing, phonological processing, spelling, and sentence processing. VLSM and other lesion-behavior approaches provide important information about which regions of the brain are critical for performance of certain language functions, but provide little insight into the brain mechanisms that may be recruited for recovery of language.

Like most other unresolved debates in aphasia recovery, the issue of the influence of lesion size and location may ultimately require large amounts of data to account for the inherent heterogeneity in stroke brains. Promising ongoing work such as the Predicting Language Outcome and Recovery After Stroke (PLORAS) database, which is a data repository of speech and language performance measures from standardized assessments and MRI scans, will provide important insights into individualized predictions of behavioral recovery based on lesion size and location information (29, 45, 46). For instance, a recent analysis of 818 stroke patients, which developed predictive models of naming scores as well-language scores, showed that disruption in connectivity between regions in the brain is associated with overall lesion location, but lesion location was sufficient to predict specific language outcomes (47). It may be that future work in this area should first focus on identifying the best methodology to delineate the singular or compounded effect of lesion location and the extent of the disruption before we can draw firm conclusions on this topic.

#### Vascular Physiology (Perfusion)

Until recently, the integrity of non-infarcted tissue has received little consideration with regard to remapping of language networks in chronic aphasia. Hypoperfusion (decreased blood flow) in acute stages of recovery has been well-studied, particularly within the ischemic penumbra, which is hypoperfused but still viable in the cortical tissue surrounding the irreversibly damaged ischemic core. Blood flow in this region is delayed more than 2 s beginning 3 to 6 h after stroke onset and lasting up to 72 h after stroke (48). If the penumbral region is reperfused within this time window it can be salvaged (49, 50). Hillis et al. have elegantly demonstrated that reperfusion of this at-risk tissue is possible, saving it from eventual fusion into the lesion core which increases lesion volume (51–55).

Importantly, recent research indicates that abnormal perfusion continues into the chronic phase of recovery (56). Even after ischemic penumbra resolution, perilesional hypoperfusion persists. Using arterial spin labeling to evaluate peak perfusion values in various parts of the brain, Richardson and colleagues (57) found a decrease in perfusion in the tissue surrounding the lesion in patients with chronic aphasia. Thompson and colleagues (58) replicated this finding, showing hypoperfusion within perilesional space, with the greatest hypoperfusion in tissue closest to the lesion. In addition, they found abnormal perfusion distal to the lesion in the left as well as in the right hemisphere. In another study, hypoperfused tissue negatively correlated with changes in blood oxygen level dependent (BOLD) signal in regions engaged to support treatment-induced language recovery (59). Nonetheless, the extent to which shifts in perfusion influence recovery of the neural networks for language is still under investigation.

#### White Matter Integrity

The degree of white matter integrity also influences the extent of behavioral recovery after stroke. Notably, Catani (60) showed that most middle cerebral artery strokes impair not only gray matter, but also extensively impair subcortical white matter tracts in the infarcted hemisphere. Hence, it is not surprising that the few published studies specifically investigating the relation between white matter tract integrity and language abilities in people with stroke-induced aphasia have found that disruption of and/or impairments in [as indicated by abnormal fractional anisotropy or signal intensity in diffusion tensor imaging (DTI)] at least some white matter tracts impact language. Specifically, relationships have been found between disruption of the left hemisphere tracts and language, i.e., between the arcuate fasciculus and speech fluency (35, 61–63), the superior longitudinal fasciculus and naming (64, 65). The integrity of the left uncinate fasciculus (UF) also may play a role, although mixed findings have been reported. Whereas, one large study found a strong relationship between the UF and object naming (64), others have not found this association (61, 65, 66). Even less studied are tracts such as the extreme capsule (that includes UF and IFOF) which also appear to be related to language skills (67, 68). It follows, then, that left hemisphere white matter integrity may be a strong predictor of language recovery following stroke (69). Indeed, as noted above, Hillis et al. noted that damage to left posterior superior temporal gyrus and superior longitudinal fasciculus and arcuate fasciculus negatively influenced naming recovery (33). In another study, integrity of the left inferior longitudinal fasciculus and left inferior fronto-occipital fasciculus was related to baseline and recovered naming skills in a group of treated patients (70). Additional studies examining the relation between white matter integrity and language impairments across language domains (and tracts) will help to clarify the impact of damaged structural connections between crucial gray matter regions on language breakdown and recovery.

The integrity of white matter tracts in the contralesional (i.e., right) hemisphere is also likely to be associated with recovery. Notably, cognitively healthy people show interhemispheric differences in white matter connections between Broca's and Wernicke's areas (i.e., the long segment of the arcuate fasciculus) (71). Based on diffusion tensor MRI (DT-MRI) and tractography analyses with 40 healthy young participants (20 males), 50% of participants showed extreme leftward lateralization, whereas, and only 17.5% showed white matter tract symmetry across the two hemispheres. They further showed sex differences in the degree of lateralization, with 85% of males, but only 40% of females, showing leftward asymmetry. First, these results highlight the high degree of variability in white matter pathway structures across individuals which critically impacts our interpretation of how white matter pathways are altered after a stroke. Second, these findings suggest that the premorbid presence and volume of right hemisphere white matter tracts likely play a role in whether or not the right hemisphere is engaged to support language in post-stroke aphasia. Forkel et al. (60) showed this in a study with 16 patients with stroke-induced aphasia during the acute stage of recovery (i.e., from 2 weeks to 6 months post-stroke). Using performance on the Revised Western Aphasia Battery [WAB-R, (72)] as an index of language ability, the results showed that the volume of the right hemisphere long segment of the arcuate fasciculus predicted language severity scores at 6 months post-stroke.

A few recent studies have examined changes in white matter connectivity as a result of language recovery (66, 73). For instance, Van Hees et al. examined changes in the generalized fractional anisotropy (GFA) values for the left hemisphere arcuate fasciculus in patients who received treatment for anomia and found that prior to treatment GFA was lower for patients relative to healthy controls but after treatment, no differences were observed between patients and controls (74). Likewise, Schlaug et al. (75) found changes in the right arcuate fasciculus in six patients with chronic aphasia resulting from a course of Melodic Intonation Therapy. Finally, Wan and colleagues showed that language therapy results in changes in the FA values in the right hemisphere, specifically in white matter regions underlying frontal, posterior superior temporal and cingulate regions (76).

In summary, even in chronic stages of recovery, lesion variables continue to influence language recovery. We have identified a few that have received the most attention in the aphasia recovery literature, but point out that there is likely an interplay between these and other factors. Further, the relation between these factors and language recovery will likely differ (a) for different areas and tracts of the brain, (b) for different language functions, and (c) as a function of lesion volume, lesion and chronological age and other factors. Future research will need to address these issues as well as how/if these and other brain variables interact with one another to impact recovery.

### Organism Extrinsic (Treatment) Factors Associated With Recovery

The fact that environmental factors affect recovery of neural networks following brain damage in both animals and humans is undisputed (8, 77–81). With respect to recovery of language networks, Thompson (82) posed questions about the role of experienced-based neural plasticity in aphasia, that is treatment-induced plasticity, in a special (millennium) issue of Brain and Language focused on key research issues for the 21st century. Among the questions posed at the time were: “does treatment influence reorganization of the language network…or does language reorganize in a…biologically predisposed manner, considering site and extent of lesion and other variables?” (p. 245). Since then, a large number of people with aphasia have been entered into studies examining this question, with results indicating that treatment does influence language reorganization in the brain, although most studies are single case reports or have included few participants, and they have not always monitored the natural history of neural change in untreated patients (i.e., in aphasic controls) or considered the reliability of repeated scans, leaving the reported results unclear. Most studies in the aphasia treatment literature also have tested the effects of naming therapy, with far fewer focused on other language impairments and no studies have examined the differential effects of treatment applied to different language domains. A strong case in support of treatment-dependent neural recovery would require this. In addition, neuroimaging methods have significantly advanced in recent years, and will continue to advance, perhaps calling into question the results of early neuroimaging studies of aphasia recovery.

The second question is far more difficult to answer. Neuroimaging studies of aphasia treatment using primarily functional magnetic resonance imaging (fMRI) have shown pre- to post-treatment changes in activation in undamaged tissue within the lesioned (usually left) hemisphere, the contralesional hemisphere, or both. A review of the aphasia treatment literature between 1996 and 2016 identified a total of 41 studies, which included 628 aphasic participants across studies (83). Of those, 90 study participants showed upregulation of neural activation in the right hemisphere (46, 59, 84–88), suggesting a transfer of language function from the left to the right, 99 participants showed increased activity in the left hemisphere (89–92) and 439 patients showed bilateral recruitment of neural tissue associated with treatment-induced language improvement (59, 93–98). In addition to increases in activation, patients in several studies also have shown downregulation of neural activation associated with successful treatment outcome, possibly reflecting increased processing efficiency (99–101).

Despite increasing evidence that therapy-induced reorganization may engage bilateral neural tissue, the best candidates for supporting language recovery in the chronic phase remains an unresolved issue. Some argue that neural tissue within the left hemisphere has greater potential to support language recovery (34). Because in most humans language is primarily processed in the left, this tissue likely has a biological capacity to support recovery. There is also a strong basis for the right hemisphere to be engaged to support language recovery, given that regions within it are engaged (albeit to a lesser extent than left hemisphere) for normal language processing in healthy adults (102), particularly as adults age (27). Reports of language recovery in left-brain-damaged patients who, after a second right-brain stroke loose the language recovered (103, 104) suggest a predisposition for language in the right hemisphere. Further, as noted above, many studies of language recovery in chronic stroke show post-treatment right hemisphere activation (96, 98, 105).

A strong theory supporting left hemisphere recruitment for recovery suggests that activation of tissue in the contralesional (right) hemisphere may be maladaptive and reflect increases in inhibitory processes exerted by the right hemisphere on the left. The excitatory/inhibitory imbalance between hemispheres have been clearly shown in early phases of recovery, with greater activation in the right hemisphere, due to decreased inhibition from the damaged left hemisphere (18). Although this asymmetry resolves in later epochs of recovery and is associated with shifts from right to left hemisphere activation, Heiss and Thiel's (106) theory of interhemispheric inhibition suggests that an imbalance between the hemispheres persists into chronic states of recovery and that right hemisphere recruitment is associated with poorer, rather than improved language performance. Studies using noninvasive brain stimulation (NIBS) have shown that inhibitory stimulation (e.g., low frequency transcranial magnetic stimulation) applied to right hemisphere regions improves language ability (107, 108), suggesting that this diminishes the inhibitory effects of the right hemisphere and enhances left hemisphere recruitment for language. Notably, however, most of these studies have shown very small changes in language [see (109), for review]. In addition, several studies using NIBS applied to the left hemisphere also have found bilateral increases in neural activation associated with language improvement (96).

Regions recruited across participants, even within language domain, vary greatly and involve left perisylvian, extrasylvian and right hemisphere homologous-region activation for various language processes. Thus, the left vs. right hemisphere debate may be an oversimplification in that recruitment of neural tissue in both hemispheres may be beneficial for recovery, which likely reflects a complex and dynamic interplay between the two. As we elaborate in the next section, resolution of these issues in future research may be best accomplished by using a network approach to study the potential neural substrates for language recovery, rather than focusing on individual regions within the language network.

To underscore this point, another line of evidence has shown activation outside of the core fronto-temporal language network during language processing in patients, including in parts of the middle frontal gyrus, the precentral gyrus, and inferior parietal cortex (98, 110–113). While in many cases activation is observed when damage to the fronto-temporal language network is substantial (110) other studies have shown activation in these domain-general regions even when frontal and/or temporal regions are spared (98, 112). Work in healthy adults has shown that these domain general regions, associated with attention, working memory, cognitive control and fluid intelligence (114), are engaged for effortful language processing (115) such as understanding or producing complex syntactic structures or ambiguous words (116, 117). Importantly, activation in these regions has been associated with the hypothesis that stroke patients recruit domain-general regions due to the increased cognitive effort required for processing language after stroke (113, 118, 119). This premise is in line with one principle of neuroplasticity—that novel functions can be assumed by tissue that was previously not engaged in those functions (9, 12). What is not yet known is whether engagement of these domain general regions, in conjunction with language regions, is associated with better language recovery in patients. Future work will need to confirm or refute this hypothesis.

## Language Network Recovery

**Figure 2:**
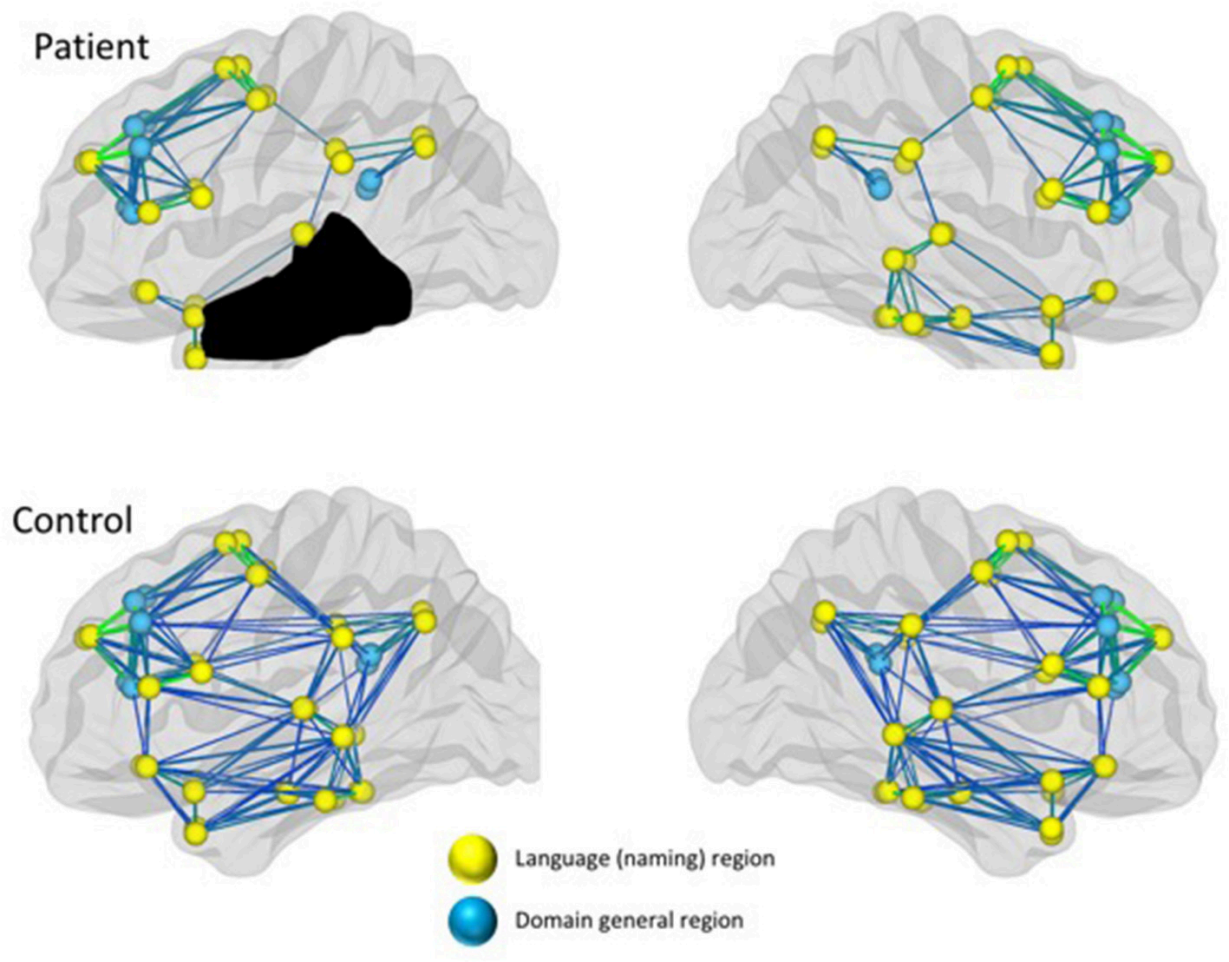
Reduced connectivity in both language and domain-general networks in a patient with aphasia compared to healthy controls.

It is now well-documented that language processing involves a complex network of left and right hemisphere regions that are often structurally and/or functionally connected (120–123). It seems reasonable to posit that language recovery engages the same complex network of left and right hemisphere regions with specific regions in the network becoming preferentially involved during the course of recovery depending on organism intrinsic and extrinsic factors, including lesion age, size, site, and treatment success. Indeed, the notion of changes in functional networks in motor and cognitive systems, altered proximally and distally by focal lesions has received substantial validation (124–127). As noted, above, language processing in PWA is also associated with activity in domain-general regions (113, 118, 119). Within the domain of language, changes in structural and functional networks also have received recent attention, examined using different approaches, including (a) structural networks (i.e., DTI), (b) task-based fMRI, and (c) resting state fMRI. Figure 2 illustrates a schematic of reduced functional connectivity (both structural and functional) in a patient with a large left temporal lesion and aphasia, compared to healthy controls, in both the left and right hemsipheres. Per the discussion above, differences in connectivity are reflected in language-specific regions as well as domain general regions. Studies also are emerging showing treatment-induced changes in connectivity (see later).

Recent work by Bonilha et al. highlights the notion that disruption of structural connectivity, that is, white matter tracts, also have far-reaching impact on language and consequently language recovery (128–132). In one study, preservation of global network integrity and, in particular, connectivity of the left middle temporal lobe was associated with greater naming treatment outcomes (34). This finding implicates the integrity of the left arcuate fasciculus as important for recovery since temporal lobe structural connectivity engages this tract. In addition, it emphasizes how network-level measures of gray and white matter regions converge to inform language recovery.

Other studies have used task-based fMRI to examine functional connectivity in language networks to understand language recovery. Examining recovery of naming, one study found changes in connectivity among four left hemisphere language areas (IFG, MTG, insula, and IPL) and their right hemisphere homologs, which were strengthened for trained but not untrained items (133). Another naming recovery study found a similar pattern, with treatment-induced network changes noted in right and left hemisphere IFG and MFG (98). Additionally, across patients, the left IFG was the most modulated region in the network as a function of rehabilitation. Another study found that the neural networks for trained and untrained items that improved with treatment showed similar changes in connectivity among language nodes. Conversely, for items that did not improve with treatment (i.e., to which generalization was not observed) the network appeared similar to networks in patients who had not been trained (111). These results indicate that networks that support training-related naming improvements differ from those engaged when naming is unsuccessful.

Task-free MRI [i.e., resting-state (rsFMRI)] data also have been used to examine the effects of brain damage on language (134–137) as well as network changes associated with recovered language. While evidence is still emerging and the studies published in this domain have utilized different approaches, all indicate that rsFMRI networks are abnormal after stroke. One study showed reduced local regional connectivity in contralesional hippocampal/para hippocampal and ipsilesional parietal and occipital regions in acute post-stroke aphasic patients (134). Another study showed hypoconnectivity across a variety of networks including attention, motor and salience networks, with the greatest reduction in connectivity within the semantic network (138). A few studies also have examined changes in resting state connectivity over time. One examined four patients who suffered posterior cerebral artery strokes over three time points (i.e., in the first week, between 3 to 5 weeks, and between 5 to 7 months) and found within and across hemisphere increases in functional connectivity associated with improved language function (139). Another study examined 14 acute stroke patients 1 and 2 months after their stroke and found that lower language comprehension was associated with lower connectivity at the first time point but at the second time point, when language skills had improved, increases in connectivity were noted (140). Improved connectivity also was reported in a study with over 100 stroke patients by Siegel and colleagues. Early post-stroke most networks in the brain were disrupted, however, the degree of local and between network integration improved over time and was associated with improved behavioral recovery (141). Similarly, Van Hees et al. (142) examined changes in amplitude of low frequency fluctuations (ALFF) (a measure of spontaneous fluctuations in fMRI signal intensity) and found reduced connectivity (correlations in ALFF) between left MTG and STG that normalized after treatment and greater connectivity between left MTG and IFG after treatment. Yet another study reported a shift in resting state modularity following treatment, compared to pre-treatment, and these changes correlated with improved language production after treatment (143). While it is premature to draw strong conclusions about what studies examining resting state networks in aphasia explicate with respect to the nature of reorganization in the brain, more work on this topic will be undoubtedly important.

To summarize, a surge in research examining the neural correlates of treatment-induced recovery, identifying regions of the brain that may be recruited to support recovery of language, and detailing both structural and functional changes in brain networks underlying reorganization have informed basic notions of neural plasticity in the aphasic brain. In due course, as this line of research becomes more and more sophisticated, it will lead to a comprehensive account or model of therapy-induced reorganization in the brain and will provide the foundation for targeted therapies to capitalize on this. Until then, in addition to intervention approaches that have been well-researched, with documentation of both the behavioral and neural effects of treatment, researchers and clinicians may rely on basic principles for promoting neural plasticity derived primarily from animal studies and research in the domain of recovery from stroke-induced motor impairments, as discussed below.

## Principles for Promoting Neural Plasticity

As highlighted earlier in this paper, results of animal studies have served to inform what we know about experience-induced plasticity following brain damage. Based on this research, Kleim and Jones (1) summarized 10 principles shown to be important for recovery that may also enhance plasticity and language reorganization in people with aphasia. Given the large literature that has accumulated since then examining the neural mechanisms of language recovery, we condense these into six basic principles relevant to aphasia treatment (see Table 1). We also add a new principle that has emerged in the aphasia treatment literature concerning complexity in language learning and recovery. We discuss these as well as the extant data and, where appropriate, approaches to aphasia treatment that support them.

**Table 1:** Principles for promoting neuroplasticity of language networks.

	**Principle for treatment of stroke-induced aphasia**	**Principles from Kleim and Jones (1)**
1	Use, improve, or lose it	Principle 1. Use it or lose it
		Principle 2. Use it and improve it
2	Specificity rebuilds targeted networks	Principle 3. Specificity
3	Salience is essential	Principle 7. Salience matters
4	Repetition and intensity promote learning and consolidation	Principle 4. Repetition matters Principle 5. Intensity matters
5	Promote generalization; avoid interference	Principle 9. Transference Principle 10. Interference
6	Complexity enhances learning and generalization	N/A

### Treatment Principle 1. Use, Improve, or Lose It

Principle 1 is based on the premises that “training that drives a specific brain function can lead to an enhancement of that function” and, conversely, that “failure to drive specific brain functions can result in functional degradation” (Kleim and Jones, p. s227). In the domain of language recovery, these principles suggest that (1) treatment focused on impaired language processes may lead to recovery of the underlying neural mechanisms associated with those processes, and (2) underuse of “specific” language systems following stroke may lead to a decrease in the ability to engage existing or new neural networks that support it. These principles argue for the use of treatment approaches that target impaired language processes.

In the domain of motor recovery, constraint induced therapy (CIT) is an approach fitting Principle 1 (144). For example, patients with unilateral upper extremity hemiparesis are provided with physical constraint of the unimpaired extremity, forcing use of the impaired limb. Some researchers have introduced a form of this for treatment for aphasia—Constraint Induced Language Therapy (CILT), which requires patients to use spoken language responses during therapy, rather than gesture, writing, or other modalities to augment communication. Although similar in principle to CIT, forcing language production *per se* may not drive specific brain functions, thus treatment may not affect specific language processes (e.g., phonological, lexical-semantic, syntactic networks). Indeed, the majority of studies of CILT have used general outcome measures of word-retrieval, repetition, and auditory comprehension as evidence for its effectiveness (66, 145–148), and although this evidence is strong, other approaches have been found to be equally or more effective (149). For example, a recent study examining the effects of CILT compared to other treatments showed that semantically-based treatment resulted in greater improvement (150). This finding suggests that treatment that targets a specific language domain may result in the strongest outcomes. This point is further discussed in the next section on the principle of treatment specificity.

### Treatment Principle 2. Specificity Rebuilds Targeted Networks

In keeping with Principle 1, Principle 2 suggests that treatment specificity rebuilds specific language networks, pointing to the use of treatment focused on primary impairments that exploit what is known about normal language representation and processing. In the aphasia treatment literature, several treatments based on psycholinguistic and cognitive neuropsychological research and models of language have emerged in recent years, which focus on improving specific language processes. In general, studies examining the effects of these treatments show positive treatment outcomes. Further, several of these impairment-based approaches also have shown neural changes associated with improved language processing, as reviewed earlier in this paper (97, 98, 105, 111).

We mention a few of these treatments here. Semantic Feature Analysis (151, 152) exploits what is known about semantic knowledge and how this knowledge connects words with one another in the mental lexicon. Similarly, phonomotor treatment (153) targets phonological networks, controlling properties of speech sounds in trained and untrained words to promote word retrieval ability. In the domain of verbs and sentence production, Verb Network Strengthening Treatment (154), focuses on improving verb production by training the lexical selection properties of verbs. Treatment of Underlying Forms (155) also makes use of the lexical properties of verbs to improve sentence comprehension and production, using a set of metalinguistic steps focused on thematic role assignment and syntactic mapping. These treatments hold promise for rebuilding language networks in stroke-induced aphasia. Research is needed to further identify the effects of these treatments on both the neural and cognitive mechanisms associated with language processing as well as the differential effects of these and other approaches. Finally, research is needed to develop and test additional treatments that exploit normal language processes.

### Treatment Principle 3. Salience Is Essential

The salience of experience also influences neural recovery. This refers to the extent to which training invokes neural systems that promote encoding, including motivation and attention. In the animal literature, studies show that when reward is coupled, for example, with an auditory tone of a particular frequency, an increase in cortical representation of that tone, but not others, occurs, indicating that motivation enhances neural plasticity (156). Attention also modulates neural activity as demonstrated in motor learning studies with both humans and animals. For example, when attention is directed away from muscle stimulation, motor encoding fails (157). This principle has not been well-explored in the context of language rehabilitation. However, several studies using fMRI have shown increased activation in domain-general mechanisms (i.e., attention/executive function) associated with treatment-induced recovery of language in patients with aphasia (118, 119). The principle of salience implies that language reorganization in aphasia may be boosted by training that couples specific treatment with methods for enhancing motivation and attention, such as using functionally significant training stimuli and/or training in functionally relevant contexts as espoused by functional- and participant-oriented treatment approaches (158, 159).

### Treatment Principle 4. Repetition and Intensity Influence Learning and Consolidation

Repetition and intensity of treatment were posed as two separate principles for promoting neuroplasticity (1). We combine these into a single principle due to the lack of a clear distinction between the two. The animal learning and sensorimotor learning literature suggest that repetition is important for facilitating neuroplasticity. Indeed, neural connections have been shown to develop through repetition, making the acquired behavior resistant to decay (160). For example, rats require repeated training trials focused on skilled-reaching behavior before measurable changes in the strength or number of synapses (161) motor map reorganization (162) are manifest. The animal learning literature also indicates the need for repeated training trials over time to facilitate maximal neural plasticity (161). Notably, principles of aphasia rehabilitation also have historically emphasized repetition. Schuell advocated “restimulation” of the language system with sufficient repetitions and providing many opportunities to respond to or produce target language behaviors both within individual treatment sessions and over time (163, 164).

The number of trials required for learning (or re-learning) and the time over which learning trials are delivered, however, is unclear. Intense training schedules have been reported to promote language recovery in aphasia, with studies indicating that higher intensity stimulation results in greater treatment gains than lower intensity stimulation (165, 166). However, the results of some other studies suggest otherwise, finding that intensive vs. less intensive treatment does not result in differential outcomes (167). Further, recent studies have shown that even though intensive treatment (i.e., massed practice) may aid with acquisition of treatment sets, maintenance of treatment effects may be boosted by non-intensive treatment, spaced over time (168, 169). Based on these studies, the benefits of intensive language treatment on facilitating optimal neuroplasticity are not yet conclusive, however, the lack of clear evidence depends partly on how intensity is defined across studies. It is unclear, for instance, whether intensity refers to the number of training hours [e.g., 2 vs. 4 h (168, 170)], the duration or spacing of training (e.g., 5 or 50 weeks of treatment with a total of 100 h) (171), or the actual number of treatment hours provided [e.g., 19 vs. 26 h (172)]. Further, studies have not explicitly addressed the content of training provided on these schedules, i.e., whether intense or spaced training schedules involve repeated training trials for improving a particular function (e.g., naming trials) or if a variety of language tasks are provided within training intervals. These differences in how intensity is quantified complicates our understanding of what ‘intensive treatment’ means and how it is related to repetition in promotion of learning and neuroplasticity. There is an emerging acknowledgment of this shortcoming (173, 174) and future studies should quantify and compare varying levels of repetition and intensity to provide more insights into this topic.

### Treatment Principle 5. Promote Generalization; Avoid Interference

Principle 5 concerns generalization, referring to the idea that “plasticity in response to training experiences can enhance the acquisition of similar behaviors” [(126); p s227]. Interference denotes the opposite effect, which potentially restricts generalization.

Generalization to untrained behaviors is an overarching goal of aphasia treatment. Indeed, aphasia treatment studies show that generalization from trained to untrained behaviors is enhanced when there is a fundamental relation between the two, for example, when trained and untrained words share common semantic, phonological, and/or orthographic features (152, 153, 175–177) or when sentences share common grammatical processes (178). These generalization patterns suggest that the same neural circuits engaged during training of target items/structures, are engaged for related untrained items.

On the flipside of this is the principle of interference: that “plasticity in response to one experience can interfere with the acquisition of other behaviors,” indicating that environmentally- or treatment-induced recruitment of, for example, non-linguistic strategies for completion of a language task may interfere with learning and generation of optimal neural networks to support linguistic processing. This principle comes mainly from studies in the motor domain showing the maladaptive effect of learned misuse (or nonuse) and its influence on motor reorganization in the brain (144, 179, 180). An emerging area of evidence for interference in the language domain comes from treatment of bilingual aphasia. In one example, a trilingual patient showed increases in interference from the trained language into the non-trained language (L2 interference to L3, and L3 interference into L2) when L2 and L3 were trained in separate 10 week phases (181). While the overall conclusion of this study impacted the nature of cognitive control in bilingual aphasia, treatment-induced cross-language interference negatively influenced the overall outcomes in the study. Future work will provide more evidence for the presence and prevalence of treatment induced interference that impacts neural plasticity.

### Treatment Principle 6. Complexity Promotes Learning and Generalization

The construct of complexity has emerged as a general principle that is relevant to treating a range of language disorders in both children and adults (177, 178, 182–184). While challenging the longstanding clinical notion that treatment should begin with simple structures, the Complexity Account of Treatment Efficacy (CATE); (185) points to the facilitative effects of using more complex structures as a starting point for treatment. In the aphasia treatment literature, the complexity effect in language learning has been found across language domains. For example, training atypical members of semantic categories (e.g., “chicken” within the category of “birds”) improves naming of typical members (e.g., “robin”) and training abstract words (e.g., “justice”) results in generalization to concrete semantically related words (e.g., “jury”) (152, 186, 187). Similarly, in the domain of morphosyntax, training complex syntactic structures (e.g., object relative or full passive) results in improved comprehension and production of less complex, linguistically related structures (e.g., object wh-questions, truncated passives, active sentences with unaccusatives verbs) (38, 59). Notably, training simpler words within lexical categories or sentence structures does not promote generalization to more complex words or structures. From a neuroplasticity perspective, these findings suggest that the neural mechanisms engaged for complex linguistic processes are also engaged for simpler ones, if/when the complex and simple material is related. Processing both abstract and concrete words, for example, engages lexical-semantic processing networks, however, concrete, but not abstract works recruit additional sensorimotor processing nodes. Thus, training abstract words within a semantic category strengthens connections within the entire network (169). Similarly, within the domain of sentence processing, complex sentences entail processes inherent in simpler, linguistically related sentence (e.g., thematic role assignment and syntactic mapping). Hence, strengthen the neural network for processing complex sentences unlocks networks required for processing simpler ones.

## Conclusion

Recovery from stroke-induced aphasia reflects a complex interplay between organism internal and external factors. The impact of nature, that is, the neural sequela of stroke is well-known to influence recovery—a non-linear process affected by neurophysiological changes in brain states from acute to chronic phases of stroke. The influence of nurture on neuroplasticity is less well-understood, however, the fact that the brain is sculpted in an experience-dependent manner throughout the lifespan argues strongly that the environment affects recovery of language networks. Emerging work has identified several organism internal and external factors that affect recovery, however, we emphasize that continued research is needed to completely understand the limits of brain malleability, particularly in chronic aphasia, and what variables are related to it.

On the nature side, we have highlighted research showing the influence of several lesion variables associated with stroke-induced language impairments, however, further research examining these and other variables as well as how, or if, they interact with one another to affect recovery is needed. The integrity of domain-general cognitive (e.g., attention, memory and executive function) and metacognitive impairments and associated neural networks also may be central to recovery and to date have received little attention in the aphasia recovery literature. Similarly, little work has focused in intrinsic psychological factors such as motivation and other personality traits. Research in these, and related domains, will be informative as to the role they play in neuroplasticity.

On the nurture side, several treatments for aphasia that exploit psycholinguistic and cognitive neuropsychological models of normal language representation and processing have been developed and studied. Indeed, these treatments show positive influences on both behavioral and neural processing. However, research in this arena is in its infancy. Research is needed not only to develop new treatments targeting specific language domains, but also to identify the differential effects of these treatments. In addition, research examining how, or if, the effects of these treatments may be enhanced, for example, by coupling non-invasive neural stimulation such as transcranial magnetic stimulation (TMS) or transcranial direct current stimulation (tDCS) with behavioral treatment, will help to inform the limits of environmental manipulations on the brain and language recovery. Finally, it is important to point out that research focused on the effects of functional social-based treatments for aphasia on neural plasticity is sorely needed. These treatments for example, may impact motivational systems, which in turn may influence language networks.

As this research unfolds, the field will move closer to developing a model of treatment-induced neural reorganization, which will provide the foundation for treatment selection and application for people with aphasia. Indeed, such a precision medicine approach is the overarching goal for clinical treatment of aphasia: to prescribe treatment and predict its outcome based on the neurocognitive deficits a patient presents, with treatment optimized for specific language deficits.

## Author Contributions

SK and CT equally contributed to the article. Both were involved in the conceptualization of the paper and in the content presented in the paper.

### Conflict of Interest Statement

SK is a scientific consultant for The Learning Corporation but there is no scientific overlap with this manuscript. The remaining author declares that the research was conducted in the absence of any commercial or financial relationships that could be construed as a potential conflict of interest.
